# Hospital and Institutional News

**Published:** 1917-12-01

**Authors:** 


### HOSPITAL AND INSTITUTIONAL NEWS.

### DOCTORS AS POLITICIANS.

It seems to be generally forgotten that M. Clémenceau, the new French Prime Minister, began life as a general practitioner in medicine. He is believed to have been fairly successful, despite certain prejudices against him founded upon the erroneous impression that he furnished his name to "L'Affaire Clémenceau," that novel of Dumas *fils* in which the hero-villain, an artist, murders his wife. In France every brilliant man seems to take to politics eventually—was not M. Berthelot a great chemist? But there it is the journalists rather than the doctors who are most frequently able to inscribe "Ancien Ministre" upon their visiting-cards. In England the doctor in politics has not hitherto been a very familiar phenomenon. It is true that Addington, the son of a medical man, was originally intended for his father's profession, and the satirists of the time were fond of calling him "The Doctor." But although we have not yet had a medical Premier, a doctor-Minister is already with us in the person of Dr. Addison, the Minister of Reconstruction, who, before he "went into" politics, was a brilliant anatomist. South Africa enjoyed a distinction which has so far been denied to the Mother-country, for in the late Sir Leander Starr Jameson, an M.D. of London, who has just died, the old Cape Colony had for four years the honour of possessing a doctor-Premier.

### THE CHILDREN'S HOSPITAL TO-DAY.

One of the hospitals which have had a curiously difficult struggle, when we consider the class of patients for whom it works, is the Queen's Hospital for Children, Hackney Road. Its work is fully appreciated in its vast district, and the Mayors of Bethnal Green, Shoreditch, Stoke Newington, and Hackney have always rallied to its support. Hospitals for children make a certain popular appeal; they used to attract legacies, but they are less fortunate than the institutions which number among their patients a recognised body of industrial workers, for behind the children there is no vested interest. There is only the congeries of slum homes. The appeal which children's hospitals make is too general to secure local maintenance, and therefore it is the duty of public-spirited men and women to come to their rescue year after year. The Queen's Hospital at this moment requires a sum of £7,000 to meet the expenses of the year. There is now but a month in which to find the money. Those who know its work realise its claim, and its circumstances, as we have shown, do not decrease its financial difficulty.

### THE TRELOAR CHILDREN'S HOSPITAL.

Preparations are rapidly being completed for the opening of two new wards at the Lord Mayor Treloar Crippled Children's Hospital, Alton, Hants, with a view to the accommodation of forty additional patients. This will bring the number of occupied beds at this hospital up to 300. The hospital at Alton is the largest in the British Empire for the treatment of tuberculosis of the bones or joints in children under the age of twelve years, and it is of outstanding importance also because of the scientifically interesting and surgically successful use which is being made there of the modern methods of heliotherapy and radiography in the treatment of this very difficult disease. From both these points of view any news of the progress of the hospital is sure to be received with widespread satisfaction. A feature of special interest in connection with the two new wards is that the beds are to be electrically heated by the method devised by the medical superintendent of the hospital after several years of experimenting. By means of this method the mattresses on which the patients lie can be heated to exactly the requisite temperature, and the heat can also be so regulated that it can be increased at the foot or middle of the bed as required, and be reduced to a minimum at the end where the head of the patient would be. It is perfectly simple, safe, and efficient, and is also very economical.

### MATERNITY HOME FOR UNMARRIED WOMEN.

Happily, people who consider an institution of this kind to be unworthy of support are now very few, and very reticent about their opinion. An appeal for support of one near London shows, however, one or two regrettable features. Passing over an excess of paper and of pietism, we note first the fact that in the appeal it was alleged that previously help had been given by one who, in fact, had never done so. This was a bad blunder to begin with, because if the reader of such an appeal finds in the first few lines something he knows to be an error he becomes immediately inspired with obstinate scepticism in the succeeding statements. In the case of one of them, at any rate, that scepticism would be justified. The lady appealer says—"The normal condition in ordinary times reveals 4 per cent. of babes born to be illegitimate. To-day this percentage has grown to 12 per cent." Now it may have grown to 12 per cent. to-day, but no one can know because statistics are not yet available. The recently-issued annual report of the Registrar General for 1915 gives the rate per 1,000 total births of illegitimate births as 44.5, or 4.5 per cent., which "compared with the rate in the quinquennium 1876-80 shows a fall of 6.3 per cent." In any case, the circular under notice is very inaccurate. It is a fact, however, that the proportion of illegitimate to total births, because of the large decline in the latter figure, is the highest since 1889. To this extent the "war baby" scare is corroborated officially.

### MEDICAL STUDENTS IN THE FRENCH ARMY.

In France all students were naturally mobilised on the outbreak of hostilities, and it is interesting to note how our Allies have dealt with the problem of maintaining an adequate supply of medical men to fill up the ranks of those who have fallen in battle. Special centres of instruction have been formed, notably at Amiens and Nancy, where students can complete their anatomical studies. At the end of each year candidates are examined by a board composed of University examiners and military surgeons, who give them certificates of proficiency. These certificates after the war will be considered as proof that the student has completed a prescribed course of study, and will thus shorten considerably the time that elapses before qualification. Similar courses in dispensing have been established at Lyons and Bordeaux, from which successful candidates pass into the Medical Corps as assistant dispensers.

### FATIGUE AND THE WORKER.

We have advanced far along the road of industrial efficiency since the days when long hours were believed to be the surest method of obtaining a maximum output. Professor Spooner, lecturing at the Munition Factory Institute at Gretna recently, gave some interesting facts showing the remarkable results that could be obtained by reducing the hours of labour, providing comfortable, well-adjusted seats for the worker, and reducing the fatigue caused by such factors as strains on the eyes and ears and compression and distortion of the feet from badly shaped and ill-balanced boots and shoes. Thus, by systematising the process, the motions of the cloth-folder were reduced from twenty or thirty to ten or twelve, whilst a typist at a contest at New York was able to beat the international champion, writing the enormous number of 147 words a minute for the entire contest, or five words a minute more than anything that had been previously done. Alcoholic beverages were to be avoided, and he recommended, as a substitute, hot water as being the only genuine stimulant for common use. The eight hours working day, he said, was the ideal for all but exceptionally strenuous trades, and workers should supplement the provisions and concessions of employers by building up their physique and vital powers. Such exercises as walking and deep breathing were invaluable in this respect. The whole question is one that merits a close study, for the nation's economic recovery from the devastating effects of the war will obviously depend upon the fullest use being made of its human resources.

### THE GROUPING OF DISTRICT COUNCILS.

Where there are a number of small local authorities anxious to adopt a progressive health policy and their size and geographical position make grouping easy, they might well follow the example set by the Urban District Council of Maesteg, in South Wales, and its neighbours. Maesteg proposed that, for the purpose of maternity and infant welfare centres, a medical officer should be jointly appointed by some half-a-dozen contiguous authorities. The suggestion has been well received, and the Bridgend Council last week signified its readiness to co-operate in the scheme, which there is little doubt will soon be carried into effect. By such grouping more efficiency can be obtained, while at the same time it leads to economy. The uniformity in the methods employed in neighbouring districts will also be beneficial.

### ORTHOPÆDIC CENTRES FOR DISCHARGED SOLDIERS.

Sir Robert Jones, C.B., Military Inspector of Orthopædics, in a recent address at the Institute of Public Health, dealt with some interesting points in the treatment of discharged soldiers. Seventy-five per cent. of men leaving an Orthopædic centre left it to go back to the Army, the remainder re-entered civil life, and it was in the endeavour to render these as efficient as possible that difficulties arose. "The returned soldier," he said, "must be dealt with very gently. He has emerged from a condition of life in which he has had only to obey and all things were done for him. He must be persuaded to take up his old independent life again, for the adult cripple becomes in so many instances a degenerate. . . Unless a wounded soldier could be made productive and an integral part of the community, it would almost be better to let him die of his wounds on the field of battle." With this end in view orthopædic centres had been or were being established in fourteen large cities, and the demand for them was steadily increasing. The choice of treatment was dependent upon the future work of the patient, and it was here that the curative workshops were of the greatest assistance, both expediting the patient's recovery and making him self-reliant and economically productive. The aim of the Pensions Ministry is to provide such centres throughout the country, where the discharged soldier, if a pensioner, can attend as an out-patient and be brought into touch with the employment bureaux, providing work suited to his particular abilities.

### THE MAN WHO "DOES NOTHING."

In showing a party of hospital workers round the 3rd London General Hospital, the well-known and energetic C.O., Colonel Bruce Porter, made the paradoxical remark that the chief officer of an institution such as that must "do nothing." He meant, of course, that he must have no hard-and-fast routine duties. Colonel Bruce Porter was undoubtedly right, for the administrator of a large establishment must be free to devote his attention to any quarter in which it may be required. He must be prepared to deal with an emergency in any direction, and to smooth away those minor difficulties which naturally occur from hour to hour in any large institution. He must also be at hand to receive any visitor of importance. It has been truly said that the busiest man is the man who has most time to spare, for the really busy man relies upon his organisation to relieve him of detail work. Yet this necessitates a staff of assistants who are absolutely dependable, and Colonel Bruce Porter, in this regard, paid a handsome tribute to his matron, his quartermaster, and the other members of his staff, without whom, he said, the 3rd London General Hospital could not have attained its present efficiency.

### SOAP FOR WOUNDS.

THE war has witnessed the introduction of a very large number of new methods of treating wounds, among which those most used are the saline method of Sir Almroth Wright, the Dakin hypochlorite solution, and the paste of Rutherford Morison, called B.I.P.P. The most recent introduction is a solution of soap, and in the *Lancet* Captain R. Garside Dixon and Captain H. T. Bates have described the method. They have employed it largely at a casualty clearing station. They did not invent the method, but learned of it from some French surgeons. At first they tried ordinary hard soap, but they found that the solution was too thick to flow along the tubes, so they employed a 2½ per cent. solution of soft soap, and this they found very satisfactory. In some cases the wound was merely dressed with sterile gauze soaked in the soap solution. The dressings were left undisturbed for three or four days, provided that there was no rise in temperature. The wound was soon clean, there was an absence of pus, and but little pain. Then they employed the solution in deeper wounds, using the Carrel tube apparatus, and it succeeded admirably. In compound fractures and in penetrating wounds of the knee-joint the results were very good, and even in cases of gas gangrene in all but one the wounds did well. The writers claim that the points in favour of the soap solution are that the wound is quickly cleaned, the dressings are much less painful than are ordinary dressings, and that as the dressings need not as a rule be changed more often than once every three or four days there is an enormous saving in labour. Further, the solution is easily procured, easily made, and cheap.

### THE CUMULATIVE ENDOWMENT OF BEDS.

THE activity of the Hospital Saturday Committee in Aberdeen has resulted in a series of periodical large gifts from a local Government works to the Nottingham General Hospital. It has therefore been decided so to alter the rules that when donations from any one body amount to over £1,000 within twelve months, the donors shall have the right to be considered as endowing a bed in the institution. Nor is this all. The endowers have the right during twenty years from the completion of the endowment to appoint a representative, who shall be privileged to recommend twelve in-patients and twenty out-patients yearly. The question of recommendations is old and thorny, and it does not grow less thorny with age. However, the "privilege" seems to be appreciated locally, and though we are not in favour of the system, the point at the moment is the recognition, in this centre, of the need, on occasion, for regarding cumulative donations as worthy of the reward attached to the endowment of a bed.

### WANTED: GERMAN SCIENCE?

REPORTS received *via* Berne of a debate in the Austrian Reichsrat reveal an appalling condition of affairs in the Dual Empire. The spread of tuberculosis and venereal disease has passed beyond all bounds. In Vienna the chairman of the Health Committee, Dr. Schacherl, admitted that the deaths from tuberculosis had almost doubled since the war. He contended, however, that medical measures were unavailing in the absence of a proper food supply, especially milk, and if, as in Vienna, the dwellings were insanitary. He stated that, at the German-Austrian Sanitary Congress in 1916, a professor had declared that eight hundred thousand soldiers were infected with venereal disease, and added that at the present time the percentage of boys in Vienna of fifteen years of age similarly infected had risen from one before the war to eight now, while in the case of youths of eighteen 27 per cent. were infected before the war and 68 per cent. now. Dr. Bobrowski, a Polish Socialist, stated that over thirty thousand persons had died in Galicia from infectious diseases resulting from war conditions. These figures are especially interesting in view of the exceptionally good health enjoyed by our own civilian population. Is German science quite powerless in matters humanitarian?

### CHESTNUTS FOR MUNITIONS.

READERS will remember that an appeal was widely circulated in the press last spring, requesting that the autumn crop of horse-chestnuts should be collected for some purpose which was not revealed. Boy and girl scouts, amongst others, organised themselves into parties for the purpose, and now a large stock of the hitherto despised "conker" is on hand. At a recent meeting of the Society of Public Analysts, an interesting paper on the subject was read by Messrs. Julian Baker and R. F. E. Hilton, in which were recorded a number of analyses of the chestnut and acorn. The chestnut contains from 21.9 to 47.8 per cent. of starch, and the acorn 55.7 to 57.1 per cent. Cane sugar was present in amounts varying from 11.7 per cent. to 19.1 per cent. in the former, and from 6.8 to 8.9 per cent. in the latter. Fermentation or hydrolysis with acid yielded between 32 to 36 gallons of absolute alcohol per ton of nuts collected. The acorn has long been used by the peasantry of many countries as a source of flour, but the bread it makes is heavy and somewhat bitter. But if it and the horse-chestnut can be utilised as a source of alcohol, much valuable food-stuff will be liberated for home consumption.

### DEATH OF A WELL-KNOWN HOSPITAL OFFICER.

THREE years after his retirement in November 1914, the death is announced of Mr. C. H. Wells, who for thirty-four years was a familiar figure at Guy's Hospital, and in the hospital world generally. Mr. Wells became clerk to the Medical School in 1880 and when the post of Secretary to the Treasurer was created (at the same time as Sir Cooper Perry's appointment as Superintendent), Mr. Wells was chosen to fill it. Mr. Wells was chiefly associated with the financial side of the hospital work, and his excellent services must have contributed substantially to the success of the great appeal which gave such impetus to the re-endowment fund. When Mr. Henry Williams died in 1913, Mr. Wells was appointed clerk to the Governors, a post which he was not destined to hold for a long period. Mr. Wells was 62 at the time of his death, and leaves a widow.

### THE ROYAL GWENT HOSPITAL.

The Royal Gwent Hospital, Newport, is finding that the various war appeals are drawing money into other channels, although the needs of the hospital are very urgent. An endeavour is being made to raise £10,000 to relieve the dire straits in which the institution is placed, and the "big push," in which Mr. W. H. Le Grand Chambers and his colleagues of the Subscription Committee are spiritedly engaged, has given exceedingly encouraging results in the first fortnight of the operations. Nearly 100 annual subscribers have undertaken to increase their subscriptions, over 100 people have been registered as new annual subscribers, and nearly 100 others have given special donations ranging from 50 guineas down to humble half-crowns. Altogether upward of £1,000 has been secured in these first two weeks.

### THE GAS-DRIVEN MOTOR-CAR.

An increasingly common street sight these last few weeks is a large object on the top of automobiles which recalls a sheeted load of hay or a small captive balloon. The scarcity of petrol has brought out this queer portable container for coal-gas, the use of compression cylinders seeming to be attended with disadvantages. How many of our readers remember a somewhat analogous appearance before laughing-gas could be packed, like an Arabian genie, into a manageably small space? Then you might see a hansom cab with an elastic bag protruding from the front of its body like a pouter pigeon, the anæsthetist peering out from behind it. On arrival at his patient's residence he would sidle through the gate, like a man with an armful of children's air-balls, to make his way through the front door and up the stairs as best he might. The present arrangement is decidedly more convenient, if not so impressive to the lay mind.

### A NEW METHOD OF ANÆSTHESIA.

New anæsthetics are not very often introduced, and even new methods of employing those already in use are sufficiently rare to justify a mention of a way of using ether as an anæsthetic which has been recently introduced. Some years ago the vapour of ether injected into the rectum was employed for the anæsthetic in operations, but the results were not on the whole satisfactory, for the ether caused no little irritation to the rectal mucous membrane, and this gave rise to a good deal of discomfort after the operation. Not long ago the rectal administration of ether had been used once more, but in a different manner. The ether is now dissolved in oil and a suitable quantity of the solution is introduced into the bowel. Usually anæsthesia is easily and rapidly produced, and it is found that a smaller amount of ether suffices than when the oral method is employed. The method is not intended apparently to replace administration by the mouth in ordinary cases, but it is of undoubted value in those instances in which it is undesirable to give ether in the ordinary way. Thus in cases in which bronchitis or any other pulmonary condition is present, the use of ether is very likely to aggravate the diseased condition of the lungs; in such cases as these the rectal channel offers real advantages. Moreover, in cases of exophthalmic goitre ether by the mouth is generally undesirable. It is clear that the rectal method is definitely contraindicated if any disease of the rectum exists. Time may show us other disadvantages, but so far the method appears to offer very real benefits in certain classes of cases.

### THE IMPOSSIBLE

If it were only possible to put in practice all the schemes which have been devised for stamping out disease, the millennium would assuredly be within a measurable distance. Unhappily, even good ideas are not always possible of accomplishment. In Parliament the President of the Local Government Board has just admitted the logic of a suggestion that cases of venereal disease in Poor-Law infirmaries should be retained till cured; but at the same time he cannot order compulsory detention because the good that would assuredly result would be counterbalanced by the evil which would follow, as many infected persons, knowing the possibility of compulsory detention, would refrain from seeking any treatment at all. A "great" idea has lately struck the Health Committee of the Barnes Urban District Council, which has directed the clerk to write to the Local Government Board asking that measures may be adopted to prevent persons suffering from ringworm and similar skin complaints of a contagious nature frequenting shops or places where food is sold. This may certainly be desirable, but the committee does not tell the Local Government Board how such measures could be enforced.

### THIS WEEK'S DRUG MARKET.

There is no sign of any probability of a general decline in the values of drugs; in fact, all the indications point the opposite way, and with few exceptions all the price fluctuations are in an upward direction. Bromides are dearer, and it is not improbable that makers of potassium bromide will advance their quotations still further; the amount of potassium bromide now being exported from the United States has reached small proportions, and the quantity available here falls short of anything like a sufficiency to meet a normal demand; the tendency is to substitute the sodium salt for the potassium salt more and more. Japanese refined camphor is dearer, and, while English refined is unchanged, the refiners are not able to sell with any freedom. Olive oil is reaching famine prices, and no supplies are expected from Spain or Italy until March at the earliest. Castor oil continues difficult to obtain even at the high prices which have to be paid for the small quantities available. Antifebrin is again dearer, as also is hexamine. The prices of sodium benzoate and benzoic acid tend upwards. Saccharin is rather more plentiful and the price is a little lower, but still in the neighbourhood of £18 a pound. Aspirin is in better supply, and the price is creeping down, but only slowly.

